# Multicenter validation of automated trajectories for selective laser amygdalohippocampectomy

**DOI:** 10.1111/epi.16307

**Published:** 2019-08-07

**Authors:** Vejay N. Vakharia, Rachel E. Sparks, Kuo Li, Aidan G. O'Keeffe, Fernando Pérez‐García, Lucas G. S. França, Andrew L. Ko, Chengyuan Wu, Joshua P. Aronson, Brett E. Youngerman, Ashwini Sharan, Guy McKhann, Sebastien Ourselin, John S. Duncan

**Affiliations:** ^1^ Department of Clinical and Experimental Epilepsy Queen Square Institute of Neurology University College London London UK; ^2^ National Hospital for Neurology and Neurosurgery London UK; ^3^ Chalfont Centre for Epilepsy London London UK; ^4^ School of Biomedical Engineering and Imaging Sciences King's College London London UK; ^5^ The First Affiliated Hospital of Xi'an, Jiaotong University Xi'an China; ^6^ Department of Statistical Science University College London London UK; ^7^ Wellcome/EPSRC Centre for Interventional and Surgical Sciences University College London London UK; ^8^ Department of Neurosurgery University of Washington Seattle Washington; ^9^ Division of Epilepsy and Neuromodulation Neurosurgery Department of Neurosurgery, Vickie and Jack Farber Institute for Neuroscience Thomas Jefferson University Hospital Philadelphia Pennsylvania; ^10^ Department of Neurosurgery Dartmouth‐Hitchcock Medical Center Lebanon New Hampshire; ^11^ Columbia University Medical Center New York New York

**Keywords:** computer‐assisted planning, laser interstitial thermal therapy, mesial temporal lobe epilepsy, selective laser amygdalohippocampectomy

## Abstract

**Objective:**

Laser interstitial thermal therapy (LITT) is a novel minimally invasive alternative to open mesial temporal resection in drug‐resistant mesial temporal lobe epilepsy (MTLE). The safety and efficacy of the procedure are dependent on the preplanned trajectory and the extent of the planned ablation achieved. Ablation of the mesial hippocampal head has been suggested to be an independent predictor of seizure freedom, whereas sparing of collateral structures is thought to result in improved neuropsychological outcomes. We aim to validate an automated trajectory planning platform against manually planned trajectories to objectively standardize the process.

**Methods:**

Using the EpiNav platform, we compare automated trajectory planning parameters derived from expert opinion and machine learning to undertake a multicenter validation against manually planned and implemented trajectories in 95 patients with MTLE. We estimate ablation volumes of regions of interest and quantify the size of the avascular corridor through the use of a risk score as a marker of safety. We also undertake blinded external expert feasibility and preference ratings.

**Results:**

Automated trajectory planning employs complex algorithms to maximize ablation of the mesial hippocampal head and amygdala, while sparing the parahippocampal gyrus. Automated trajectories resulted in significantly lower calculated risk scores and greater amygdala ablation percentage, whereas overall hippocampal ablation percentage did not differ significantly. In addition, estimated damage to collateral structures was reduced. Blinded external expert raters were significantly more likely to prefer automated to manually planned trajectories.

**Significance:**

Retrospective studies of automated trajectory planning show much promise in improving safety parameters and ablation volumes during LITT for MTLE. Multicenter validation provides evidence that the algorithm is robust, and blinded external expert ratings indicate that the trajectories are clinically feasible. Prospective validation studies are now required to determine if automated trajectories translate into improved seizure freedom rates and reduced neuropsychological deficits.

**Key Points**

- Computer‐assisted planning for laser trajectories may provide an automated means of optimizing and standardizing ablation volumes
- We undertake a multicenter external feasibility study of manual and automated trajectories to compare trajectory parameters and estimated ablation volumes
- External blinded surgeons were significantly more likely to give preference to automated compared to manually generated trajectories
- Automated trajectories returned significantly improved calculated risk scores and amygdala ablation (%), while reducing ablation of collateral structures
- Prospective studies are required to determine if the improved trajectory parameters and ablation volumes translate into clinical benefit

## 1 INTRODUCTION

Laser interstitial thermal therapy (LITT) is a novel minimally invasive technique for performing highly selective ablations within the brain^1^ and spine.^2^ Contemporary series of LITT for mesial temporal lobe epilepsy (MTLE) have reported seizure‐freedom rates comparable to those for open neurosurgical resection,^3^, ^4^, ^5^ with some centers now offering this as first‐line treatment.^6^ The highly selective nature of the thermal ablation has also been suggested to result in less neuropsychological deficit as a consequence of limiting damage to collateral brain structures.^7^, ^8^ LITT delivered through either the Visualase (Medtronic Inc.) or NeuroBlate (Monteris Medical) system involves the stereotactic placement of a laser fiber within the region of interest (ROI) through a small craniostomy along a predefined trajectory.^9^ The extent of the ablation and heat dissipation to surrounding brain structures is monitored using magnetic resonance (MR) thermography.^10^ LITT results in ablation diameters of 5‐20 mm in diameter and is susceptible to heat sinks such as cerebrospinal fluid (CSF) cavities and vasculature.^11^ The safety and efficacy of LITT for MTLE is dependent on the preplanned trajectory and the volume of the thermal ablation. To date, only a single study has suggested that the extent of the mesial hippocampal head ablation is an independent predictor of seizure‐free outcome.^12^ The enthusiasm for LITT, however, must be tempered as overall reported complication rates are between 5% and 22.4%,^13^ including intracranial hemorrhage, visual field deficit, and cranial neuropathy. Given the lack of evidence defining optimal ablation parameters for a seizure‐free outcome, neurosurgeons apply a number of heuristics when planning LITT trajectories.^9^ These include identifying an avascular corridor for the laser catheter, avoidance of ventricles and sulci where possible, ablation of the hippocampus back to the level of the tectum, and maximizing distance from critical structures such as the brainstem and lateral geniculate nucleus.^9^, ^14^, ^15^ In addition, minimizing the intracerebral length of the trajectory and drilling angle to the skull may reduce parenchymal transgression and implantation inaccuracy, respectively.

We have previously derived LITT trajectory parameters from expert opinion and incorporated this into the EpiNav (King's College London) software platform to automate LITT trajectory parameters.^14^ We demonstrated improved trajectory metrics including estimated ablation volumes of the amygdalohippocampal complex (AHC) over manually planned trajectories. We next applied a machine learning approach to deriving the LITT trajectory parameters that further increases the estimated ablation of the AHC, while sparing ablation of the parahippocampal gyrus (PHG).^16^ This revealed that optimal entry points cluster around the temporo‐occipital junction and optimal target points are at the anterior medial aspect of the amygdala. However, it is unclear if these machine learnt trajectories are generalizable to external surgeons with varied planning practices.

In this study we perform a multicenter validation of automated (both expert‐derived and machine‐learnt parameters) using the approach above, vs manually planned trajectories for MTLE LITT. Comparators include trajectory metrics, estimated ablation volumes, and external blinded feasibility ratings.

## 2 METHODS

This article was prepared in accordance with the strengthening the reporting of observational studies in epidemiology statement.^17^

### 2.1 Patient inclusion

Ninety‐five patients from three high‐volume epilepsy surgery services (Thomas Jefferson University Hospital, n = 25; Harbor View Medical Center, n = 48; and Columbia University Medical Center n = 22) were included in this multicenter validation study following a prospective power calculation (see Section 2.6). Each center has a large series and established expertise in using LITT for MTLE. Consecutive patients were included if they had received LITT for MTLE and had concordant semiology, scalp electroencephalography (EEG) and structural magnetic resonance imaging (MRI) features of mesial temporal sclerosis, or had seizure onset confirmed within the hippocampus following stereo‐EEG (SEEG) investigation. Ethical approval for the study was provided by institutional review board approval at each of the collaborating institutions for the retrospective use of anonymized imaging: (Thomas Jefferson University Hospital: #15D.106, Harbor View Medical Center: #STUDY0006292, Columbia University Medical Center: #AAAS5654.)

### 2.2 Manual trajectory generation

Implemented laser catheter trajectories were determined from the postoperative MRI scans from three different LITT centers. Postoperative T1 MR images were registered to the preoperative MRI scans within EpiNav using a rigid transformation^18^ and manually checked to ensure accurate registration. In cases where rigid registration failed, due to an insufficient field of view, a landmark registration using the anterior and posterior commissures was applied. Implemented (manually planned) trajectories were then reconstructed by manual selection of the entry and target points on the postoperative T1 MRI scans. Manual trajectories were denoted as *Trajectory 1*.

### 2.3 Automated trajectory generation

**Figure 1:**
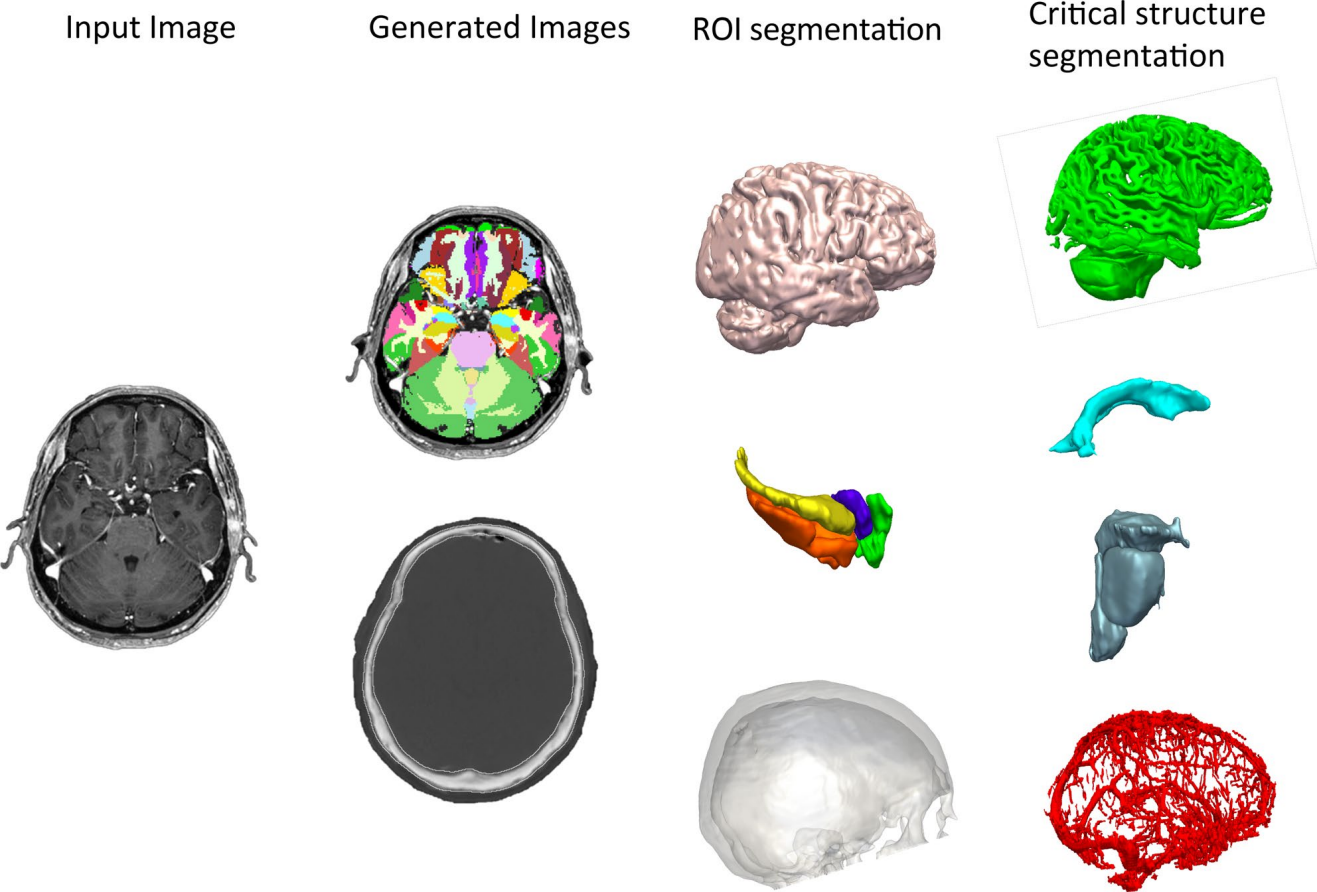
Summary of model generation. Input image consists of a single volumetric gadolinium‐enhanced T1 image from which a whole‐brain parcellation and pseudo‐CT image are generated. Regions of interest segmented from the geodesic information flows (GIF) parcellation include the cortical surface, hippocampus (yellow), amygdala (purple), parahippocampal gyrus (orange), and entorhinal cortex (green). Skull model segmented from the pseudo‐CT. Critical structures segmented from GIF parcellation include sulci, lateral ventricles, and brainstem. Vascular segmentation from gadolinium‐enhanced T1 image following application of a Sato filter^37^

Computer‐assisted planning (CAP) for automated generation of LITT trajectories using EpiNav was performed prior to assessment of the manually implemented trajectory and has been described previously. We refer readers to our previous work for a more in‐depth description of the CAP algorithm.^14^, ^16^ In brief, a preoperative T1 image is used to generate a patient‐specific whole‐brain parcellation^19^ and pseudoCT^20^ using geodesic information flows (GIFs). The corresponding whole‐brain parcellation is then used to derive models of the cerebral cortex, inferior occipital gyrus, lateral ventricles, sulci, brainstem, amygdala, hippocampus, entorhinal cortex, and parahippocampal gyrus (see Figure 1). The lateral ventricle was defined as a no‐entry zone, whereas vasculature, brainstem segmentation, and sulcal models were included as critical structures. Minimum distance from critical structures was set at 3 mm.^21^ Trajectories were limited to a maximum length of 120 mm, to prevent excess parenchyma transgression, and a drilling angle orthogonal to the skull of <30 degrees to prevent skiving at the bone during drilling.^14^ Minimum distance to the brainstem was set to 7 mm for all automated trajectories to prevent excess heat transmission.

In this study, we compare clinical feasibility and estimated ablation volumes for three automated trajectories based on our previous work. The first was constrained to entry through the inferior occipital gyrus targeting the centroid of the amygdala (denoted as *Trajectory 2*). This represents the benchmark parameter for automated planning and most closely replicates the manual planning parameters described by Wu et al.^9^ The second incorporates the trajectory parameters derived from expert consensus^14^ and incorporates an entry point through the inferior occipital gyrus targeting a 3‐mm anterior, medial, and inferior translation of the centroid of the amygdala (denoted as *Trajectory 3*). Finally, the third trajectory employs trajectory parameters derived from machine learning.^16^ These include an entry region at the temporooccipital junction, targeting a 3‐mm anterior and medial translation of the centroid of the amygdala without an inferior translation (denoted as *Trajectory 4*). See Figure 4B and D in Li et al (2019)^16^ for more details.

### 2.4 Trajectory parameter analysis

**Figure 2:**
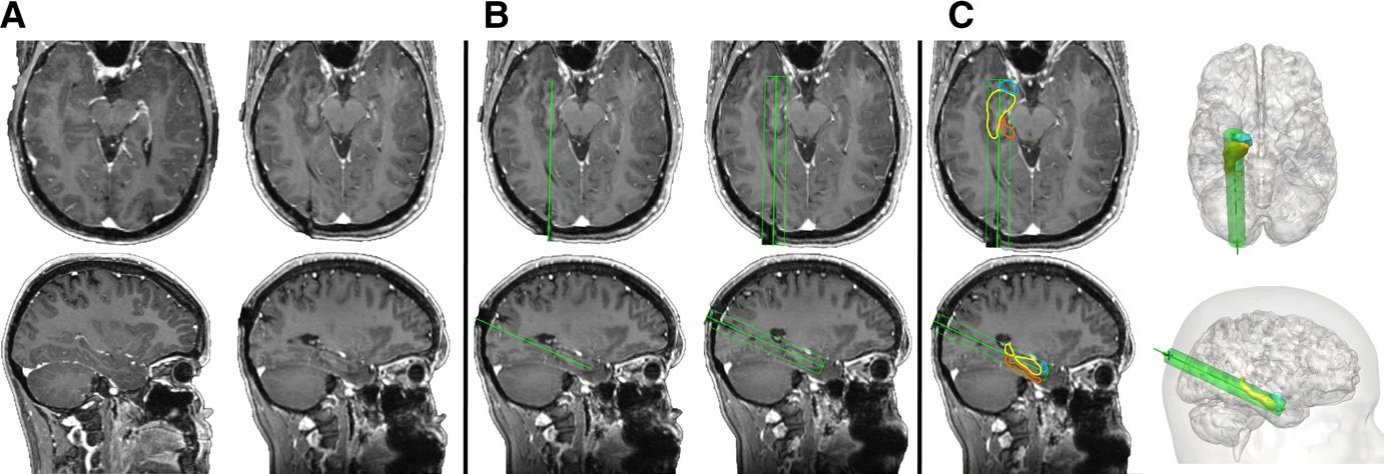
A, Axial and sagittal images of pre‐ (left) and post‐LITT ablation (right) in the same patient. B, Manual trajectory recreated from post‐LITT ablation (left) and with estimated maximal 15‐mm diameter uniform ablation cavity applied (right). C, Overlap of estimated ablation cavity with regions of interest (amygdala, blue; hippocampus, yellow; and parahippocampal gyrus, orange) used to calculate ablation volume and also shown on the three‐dimensional (3D) model (right). Note: Parahippocampal gyrus not shown on the 3D models for clarity

For each of the four corresponding trajectories (one manual and three automated) per patient, the calculated trajectory parameters were calculated and returned in an automated fashion. Calculation of the risk score has been described previously^22^ and provides a numerical representation for the size of the avascular corridor. In brief, the risk score is calculated by assigning 128 nodes along the trajectory and measuring the distance from vasculature at each of the indices. The same number of nodes are assigned regardless of the trajectory length to prevent longer trajectories from accruing greater risk scores. The risk score is then normalized to provide a score of >1 if the trajectory to vessel distance was less than the user‐defined safety margin (3 mm in this case). A uniform 5‐15 mm diameter ablation zone^14^ (see Figure 2) was then applied to each trajectory and the volume of overlap with the amygdala, hippocampus, entorhinal cortex, and parahippocampal gyrus, as determined from the brain parcellation, was automatically calculated in each case. The estimated ablation volumes were normalized by the preoperative volume to provide the percentage of ablation for each structure.

### 2.5 Expert ratings

Three expert neurosurgeons were selected as blinded raters. Each was provided with four trajectories from 23 randomly selected patients (92 trajectory ratings in total). To assess interrater variability, the first 16 randomly selected patients were sent to all raters and the remainder of patients were uniquely assigned. Raters were blinded to the trajectory generation method and were asked to rate the feasibility of the entry, trajectory, and target points of the four laser trajectories. Feasibility criteria were based on whether the expert raters would be willing to implant the trajectory as part of their current surgical practice. In addition, raters were asked to rank the blinded trajectories in order of preference from 1 to 4, with 1 being the most and 4 the least preferred.

### 2.6 Statistical analysis

Trajectory parameters for the four trajectories per patient were analyzed using an analysis of variance (ANOVA) with post hoc pair‐wise comparisons and Bonferroni correction for multiple comparisons.

Expert ratings between three independent neurosurgeons were assessed using a mixed‐effects logistic regression model estimating the binomial probability distribution of trajectory feasibility (=1) compared to infeasibility (=0). Cohen's Kappa statistic for pairwise assessments between the expert raters was also performed. Analysis of expert preference was performed using Pearson's χ^2^ and an ordinal logistic regression model. Statistical analysis was performed using SPSS 25 (IBM Corp.) and Stata Statistical Software: Release 15 (StataCorp LLC.)

A prospective sample size calculation was performed to determine the number of patients to sample to be able to detect a reduction in risk score of at least 0.2 units using a two‐sample *t* test with a significance level of 1% and a power of 90%. In performing this calculation, it was assumed that the standard deviation of risk score is 0.25 (estimates derived from pilot data and previous work). In total, 92 patients would need to be recruited to achieve this. This sample size would also allow detection of at least 10% difference in ablation volumes at a power of 0.9 given a 0.14 standard deviation.

## 3 Results

### 3.1 Trajectory metrics

Mean trajectory parameters for the four calculated trajectories (one manual and three automated) are shown in Table 1. A one‐way ANOVA model suggested a significant difference in the mean planned trajectory length, drilling angle to bone, and risk score (*P *<* *.0001). Post hoc analysis with Bonferroni correction (see Table S1) revealed that manual planned trajectories had significantly longer trajectory lengths and greater risk scores compared to the automated trajectories (1 vs 2‐4). Drilling angle to the skull was significantly less (ie, more orthogonal to the bone), with trajectories 1‐3 compared to trajectory 4.

**Table 1:** Comparison of trajectory metrics between different trajectory generation methods

	1 (Manual)	2 (Automated centroid of amygdala)	3 (Automated anteroinferior mesial amygdala)	4 (Automated anteromesial amygdala)	Statistical significance (ANOVA model)
Length (mm)	103.6 ± 10.0	93.5 ± 8.4	95.8 ± 8.2	89.0 ± 7.4	*P *<* *.000*
Angle (deg)	29.3 ± 6.5	28.8 ± 6.8	28.9 ± 6.2	31.8 ± 6.0	*P *=* *.003*
Risk score	1.3 ± 0.1	1.1 ± 0.2	1.1 ± 0.2	1.1 ± 0.2	*P *<* *.000*
Brainstem distance (mm)	7.3 ± 2.4	6.7 ± 2.3	6.5 ± 2.1	7.0 ± 2.1	*P *=* *.053

*Denoted statistical significance following correction for multiple comparisons.

### 3.2 Ablation volumes

A one‐way ANOVA revealed a significant difference in the mean ablation length, total ablation volume, amygdala ablation (%), hippocampal ablation (%), entorhinal cortex ablation (%), and parahippocampal gyrus ablation (%) (see Table 2) between trajectories. Post hoc analysis with Bonferroni correction (see Table S1) revealed that manual trajectories had a significantly shorter ablation length (mm) compared to the automated trajectories (1 vs 3; 1 vs 4). Manual trajectories also had a significantly greater total ablation volume (1 vs 2; 1 vs 4) but achieved a significantly smaller percent ablation of the amygdala (1 vs 3; 1vs 4) and hippocampus (1 vs 4). Finally, manual trajectories resulted in a significantly greater percent ablation of the entorhinal cortex (1 vs 2; 1 vs 3; 1 vs 4) and parahippocampal gyrus (1 vs 2; 1 vs 4).

**Table 2:** Comparison of ablation parameters between different trajectory generation methods

	1 (Manual)	2 (Automated centroid of amygdala)	3 (Automated anteroinferior mesial amygdala)	4 (Automated anteromesial amygdala)	Statistical significance (ANOVA model)
Ablation Length (mm)	24.9 ± 11.0	27.1 ± 6.9	30.4 ± 6.8	28.6 ± 7.7	*P *<* *0.000*
Ablation volume (mm^3^)	3535.7 ± 1021.4	3021.1 ± 906.3	3630.4 ± 830.9	3203.9 ± 998.6	*P *<* *0.000*
Amygdala ablation (%)	45.3 ± 22.2	44.5 ± 16.2	58.7 ± 14.0	64.2 ± 20	*P *<* *0.000*
Hippocampal ablation (%)	67.3 ± 16.3	65.2 ± 14.5	67.9 ± 12.8	61.6 ± 13.8	*P *<* *0.012*
Entorhinal cortex ablation (%)	17.8 ± 18.0	2.1 ± 4.4	7.2 ± 8.1	8.7 ± 7.7	*P *<* *0.000*
Parahippocampal ablation (%)	25.1 ± 17.9	17.1 ± 14.00	28.3 ± 17.8	11.0 ± 11.6	*P *=* *0.000*

*Denoted statistical significance following correction for multiple comparisons.

### 3.3 Feasibility ratings

Mixed‐effects logistic regression models (Table S3) were applied to subjects, with a random effect to account for clustering of trajectories within patients, in whom ratings were provided by all three raters and revealed a significant difference between trajectory generation methods, despite correction for the significant difference between raters (test statistic = 59.61, 2 d.f., *P *<* *.01). Overall, automated trajectories 2‐4 were significantly more likely to be rated as feasible by external raters (test statistic = 24.21, 3 d.f., *P *<* *.01), despite the manual trajectories having been stereotactically implanted in all patients.

**Figure 3:**
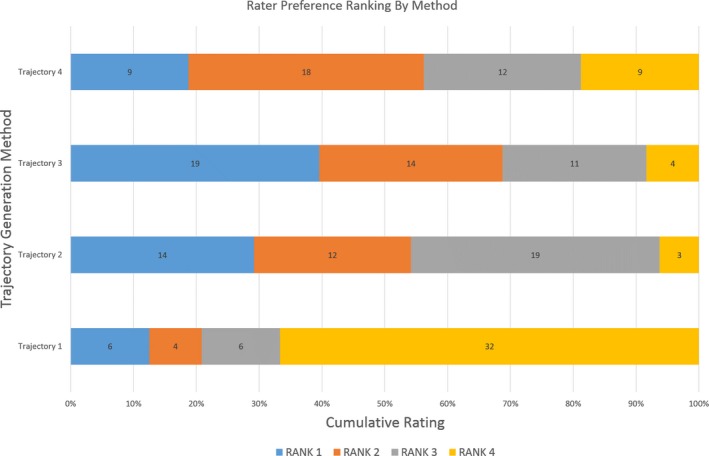
Summary of blinded expert rater preferences (rank 1‐4) by trajectory generation method (1‐4). Trajectory 1 (manually planned) was significantly more like to be ranked fourth (least favorable) compared to trajectories 2‐4 (automated)

Comparison of ordinal rater preferences between trajectory generation methods revealed “fair” agreement^23^ between all raters with a Cohen's Kappa statistic of 0.333 (rater 1 vs rater 2), 0.235 (rater 1 vs rater 3), and 0.333 (rater 2 vs rater 3). Pearson's χ^2^ analysis revealed a significant difference (*P *<* *.001) between the observed and expected distribution of expert preference ratings between trajectory generation methods. Ordinal logistic regression was then performed to examine if there was any difference between trajectory preference ratings after accounting for raters. This revealed that the manual trajectories (method 1) had the greatest probability of being assigned the lowest rater preference, that is, preference 4 (Table S4 and Figure 3).

## 4 Discussion

### 4.1 Key results

LITT trajectory planning requires optimization of a number of complex parameters including trajectory metrics for safety and ROI ablation for seizure freedom and neuropsychological outcome.^3^, ^4^, ^7^, ^24^ We present a multicenter validation of different automatically generated stereotactic trajectories using the EpiNav platform and compare these to manually planned (implemented) trajectories from three different institutions. In addition, we provide trajectory feasibility ratings from three external blinded experts. We find that automated trajectories are significantly shorter and have improved risk scores. We also show that automated trajectories have increased ablation length and amygdala ablation (%) with decreased parahippocampal gyrus ablation (%). External blinded experts were significantly more likely to prefer and rate automated trajectories (2‐4) as feasible.

### 4.2 Summary of LITT studies to date

Single‐center case series report that LITT for MTLE is a safe and effective first‐line alternative to open temporal lobe surgery in cases of mesial temporal sclerosis or where mesial temporal seizure onset has been proven by SEEG.^3^, ^4^, ^5^, ^6^, ^12^, ^24^, ^25^ A recent meta‐analysis of LITT for MTLE has suggested an overall seizure freedom rate of 50% at 12‐36 months, rising to 62% when considering “lesional” cases only.^26^ Despite a slightly lower seizure freedom rate compared to open surgery, the minimally invasive nature of the procedure and superior patient satisfaction makes LITT an attractive first‐line alternative. To date, there have not been any randomized comparisons of LITT to open surgery, but a prospective parallel‐group study has shown superior postoperative object recognition and naming following LITT.^7^ The focal nature of the ablation and lack of damage to the surrounding critical structures and temporal neocortex have been suggested as possible reasons for this. The Feasibility Study on Laser Interstitial Thermal Therapy Ablation for the Treatment of Medical Refractory Epilepsy (FLARE) (ClinicalTrials.gov Identifier: NCT02820740) and Stereotactic Laser Ablation for Temporal Lobe Epilepsy (SLATE) (ClinicalTrials.gov Identifier: NCT02844465) are two open‐labeled prospective studies from Monteris Medical and Medtronic Inc., respectively, that are currently ongoing. As with any novel technology, there is an associated learning curve, and it is possible, therefore, that early case series may both underestimate the therapeutic potential of LITT and overestimate risks.

Despite the promise of LITT as an alternative first‐line therapy, a single case series has reported an overall complication rate of 22.4%,^27^ including catheter misplacement, intracranial hemorrhage, device malfunction, hemiparesis, cranial neuropathy, and visual field deficits.^4^, ^13^, ^14^, ^27^, ^28^ Intracranial hemorrhage may be due to catheter misplacement resulting in an unplanned conflict with a vessel, inability to visualize small vessels on radiographic images, or planning trajectories through avascular corridors that are too restrictive. To this end, we calculate “risk score” as a mathematical representation of the size of the avascular corridor for the entire planned trajectory. Due to the complexity of the calculation, it is not possible for the surgeon to calculate this during manual planning and as such they must depend on their experience to estimate this. Intraventricular hemorrhage was described in one of three cases of LITT for MTLE in the series by Pruitt et al.^27^ Trajectories passing through the ventricles are therefore avoided where possible due to the risk of intraventricular hemorrhage, CSF leak, and potential heat sink effect, as recommended from the earliest series.^15^

**Figure 4:**
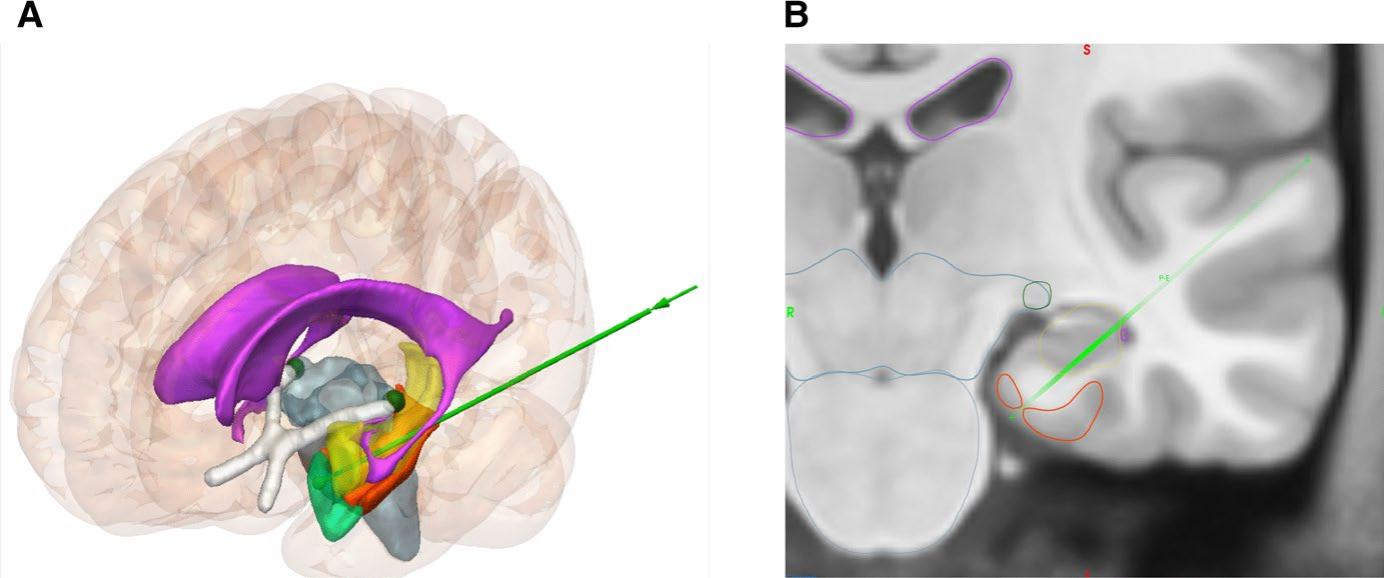
Template‐based A, 3D model and B, coronal MRI of an automated left sided LITT trajectory (light green) at the level of the LGN (dark green) showing its incorporation within the brainstem segmentation (blue). Entire LITT trajectory overlaid onto 2D coronal slice with intrahippocampal (opaque green) and extrahippocampal (transparent green) portions delineating the lateral to medial and superior to inferior angulation of the trajectory. Position of trajectory at level of LGN (green). Amygdalohippocampal complex (yellow), lateral ventricles (pink), parahippocampal gyrus (orange), entorhinal cortex (teal), and optic tract (white) also shown

Visual field deficits (VFDs) are the most common complication associated with LITT for MTLE and include contralateral superior quadrantanopia and hemianopia.^4^ Anatomically, superior quadrantanopia results from ablation cavities extending posterior to the hippocampus into the optic radiation posterolaterally within the sagittal striatum,^4^ whereas homonymous hemianopia may result from heat transfer to the lateral geniculate nucleus (LGN) during posterior hippocampal ablation.^13^ EpiNav‐generated trajectories run inferior to the lateral ventricle and therefore prevent excessive heat transfer to the sagittal striatum (lateral to the body of the hippocampus) so they are less likely to result in superior quadrantanopia secondary to optic radiation injury. The ambient cistern and choroidal fissure separate the body of the hippocampus from the ventral diencephalon (superiorly) and brainstem (medially), respectively. A single study has suggested that patients at most risk of heat transfer to the LGN, and therefore homonymous hemianopia, are those with low choroidal fissure CSF volume. EpiNav maximizes distance from the brainstem, based on a user‐specified parameter, in order to prevent thermal injury. The LGN is included within the brainstem segmentation and represents the most lateral aspect. Through maximizing the distance from the brainstem, EpiNav automatically maximizes distance from the LGN (see Figure 4).

Transient cranial neuropathies (oculomotor and trochlear nerves) have been reported and are thought to result from heat transfer medially at the tentorium during ablation of the mesial hippocampal head and entorhinal cortex.^4^, ^14^ We have previously shown that EpiNav‐generated trajectories maximize ablation of the mesial hippocampal head,^14^ as this has also been suggested to be an independent predictor of postoperative seizure freedom.^12^ Extra care will be required, therefore, when implementing EpiNav‐generated trajectories, as these patients may be at a theoretically higher risk of this complication.

### 4.3 Interpretation of results

#### 4.3.1 Ablation parameters

The largest outcome study to date, analyzing MTLE LITT ablation outcomes in 234 patients from 11 centers, has revealed that greater volumes of amygdala ablation are more likely to be associated with seizure‐free outcomes, whereas extensive hippocampal ablations, with more posterior hippocampal extension, are counterproductive.^29^ Other regions implicated in improving seizure freedom based on the construction of voxel‐wise positive predictive value maps include more mesial‐, anterior‐, and inferior‐based ablation cavities. Prior to this study, only the extent of mesial hippocampal head ablation had been shown to correlate with seizure‐free outcome in a single‐center case series,^12^ but this finding has not been replicated in larger series. In line with these studies the automated trajectories significantly improved amygdala ablation volume and spared the posterior hippocampus compared to the manually planned and implemented trajectories, suggesting that automated trajectories may improve seizure‐free outcomes and potentially spare neuropsychological function, but prospective validation will be required to prove this.

Typically laser ablations of the amygdala and hippocampus include varying amounts of surrounding structures such as the entorhinal cortex and white matter tracts.^4^ This suggests a complex correlation that may be affected by other, as yet undefined, ablation parameters as well as patient selection. Furthermore, the total ablation volume of the hippocampal ablation does not correlate with seizure‐freedom rate, despite correction for preoperative anatomic volume.^3^, ^4^, ^14^ The extent of hippocampal resection in the open surgical literature has also failed to show a significant correlation with seizure‐freedom rates in MTLE.^30^ We hypothesize therefore that there is a critical volume of amygdala and hippocampal ablation or resection that encompasses the seizure‐onset zone resulting in seizure freedom. Given that this is likely to be patient specific and no study has been able to estimate this, the EpiNav‐generated trajectories maximize ablation of the hippocampal head and body, while sparing the tail by virtue of the more lateral entry and more medial target point. The hippocampal tail has been suggested to be important for the preservation of episodic memory following open surgical resections^31^, ^32^ (see Figure S1). EpiNav (trajectories 3 and 4) resulted in a significantly greater ablation of the amygdala compared to manually planned trajectories, with significantly improved risk scores (trajectories 2, 3, and 4) and only a small reduction in hippocampal ablation (trajectory 4). The parahippocampal gyrus ablation was also significantly reduced (trajectory 2 and 4), which has been suggested as a possible reason that laser ablation results in retained neuropsychological function compared to open surgical resection.^8^, ^33^, ^34^, ^35^

#### 4.3.2 External Expert Feasibility Ratings

We undertook a multicenter retrospective validation of the algorithm to do the following: (a) ensure that the algorithm is generalizable across centers, (b) maintain performance with imaging from different institutions, and (c) compare the algorithm to a variety of planning practices from different neurosurgeons. External expert raters were blinded to the trajectory generation methods and asked to rate the entry, trajectory, and target feasibility individually. After reviewing the 4 trajectories per patient, the raters then ordered them based on preference according to their current clinical practice. The trajectory parameters, including ablation volumes and risk scores, were not provided to the external experts so as not to bias the ratings. There was significant variation between the ratings provided by the external blinded raters for trajectory feasibility, whereas ordinal ratings between trajectory preference rankings showed fair correlation. When correcting for the difference between surgeons we found that automated trajectories were significantly more likely to be rated as preferred compared to manual trajectories. Given that the EpiNav platform optimizes trajectory heuristics that are normally considered by neurosurgeons during planning, we hypothesize that this is why automated trajectories are more broadly acceptable. In addition, trajectory 3 was used in our initial comparative study^14^ and was derived from expert consensus. By comparison, trajectory 4 entry was defined solely through machine‐learning parameters.^16^ Taking into account the trajectory safety metrics, ablation volume estimations, and external expert feasibility ratings, we would advocate providing the surgeon with a choice of trajectories 3 and 4 for use in future prospective validation studies.

### 4.4 Generalizability

As more centers perform LITT for MTLE, each will face a learning curve that may result in increased complication rates and poorer seizure‐free outcomes until this is overcome. Automated trajectory algorithms optimize planning parameters in an objective and systematic fashion based on user‐defined parameters.^14^ The EpiNav platform is based on the current literature to date and therefore benefits from the combined learning curves of multiple centers as well as the incorporation of machine learning parameters.^16^ As further data are acquired, the algorithm is adaptable to continually incorporate and optimized these features. Consistent planning strategies across institutions are key to ensuring standardized outcomes and reducing patient morbidity.

## 5 LIMITATIONS

The main limitation of this work is that it is a retrospective comparison of simulated EpiNav automated trajectories to manually planned and implemented trajectories in the same patients. As such, we do not have the actual parameters that would be achieved if the automated trajectories were implemented or the seizure freedom and neuropsychological outcomes associated with the automated trajectories. A retrospective analysis based purely on calculated trajectory parameters does, however, allow different trajectories to be modeled and compared in the same patient, allowing for direct comparisons between trajectory metrics. In addition, we have shown previously that the estimated ablation volumes accurately reflect that which were achieved following ablation,^14^ which relies on the assumption of a uniform 5‐15 mm diameter ablation cavity. Further studies of laser ablation dynamics have also corroborated this to be the case in both the axial and sagittal dimensions of the ablation cavity (see Figure 3 in reference 11^11^). We acknowledge that thermal ablation patterns are nonlinear and vary significantly between patients, but we are currently unable to model this complexity on an individual patient basis. To prevent systematic bias, comparisons between the manual and automated trajectories were based on the same calculated ablation volumes. In addition, although EpiNav provides a trajectory and resultant simulated ablation cavity, a highly skilled surgeon is required to interpret the MR thermography and accurately contour the ablation cavity to prevent damage to collateral structures.

A further limitation is that the automated trajectory generation pipeline within EpiNav is based on the use of a whole brain parcellation algorithm. In this study, we used GIF,^19^ but the pipeline can be used with other parcellation methods including Freesurfer.^36^ Given that the whole‐brain parcellations are developed and typically validated on brain MRI scans from healthy controls, it is likely that they may oversegment regions of atrophy, such as the hippocampus in patients with severe mesial temporal sclerosis. To mitigate this effect, the whole‐brain parcellations were manually checked for accuracy in all cases and the same ROI segmentations were used for all ablation volume comparisons.

Comparison of trajectory feasibility ratings and preference order between the external expert raters showed significant differences and only fair correlation, respectively. Given the variety in surgical practices associated with LITT trajectory planning, we implemented a mixed‐effects logistic regression model and have shown that the differences in trajectory feasibility ratings between methods were still significant despite accounting for the differences between the expert raters.

EpiNav LITT trajectories currently plan ablations based on a single trajectory. We acknowledge that in some circumstances more than one trajectory may be required to achieve a technically successful ablation.

## 6 CONCLUSIONS

Building on our previous work, we provide a large multicenter validation study of 95 patients across three neurosurgical centers. To our knowledge, this is the largest validation of an automated trajectory planning system to date. We show that the algorithm is robust to different MRI acquisition parameters and that all automated trajectories were consistently given higher preference ratings compared to the manual trajectory. Automated trajectories significantly improved percentage of amygdala ablation, which has been found to correlate with seizure freedom. These results justify a prospective validation study to determine if the automated trajectory metrics and estimated ablation parameters result in improved safety, seizure freedom rates, and reduced neuropsychological morbidity compared to current manual planning. Future work should also aim to integrate diffusion‐weighted imaging to consider critical white matter fiber tracts that are important for visual and neuropsychological function. Although not available currently, nonlinear estimations of the expected ablation cavity would also enhance this work. A prospective validation study in which the automated trajectories are implanted is the focus of future work.

## CONFLICT OF INTERESTS

Relevant to this work, C.W. is a paid consultant for Medtronic, NeuroPace, and Nevro, and is on the advisory board for Microsystems Engineering, Inc., but these are not related to this work. The remaining authors have no conflict of interests. We confirm that we have read the Journal's position on issues involved in ethical publication and affirm that this report is consistent with those guidelines.
