# Supplementary figures and images for: Multicenter validation of automated trajectories for selective laser amygdalohippocampectomy
Source: Epilepsia. 2019 Aug 7;60(9):1949–59. doi: 10.1111/epi.16307 (PMC6771574; doi:10.1111/epi.16307)

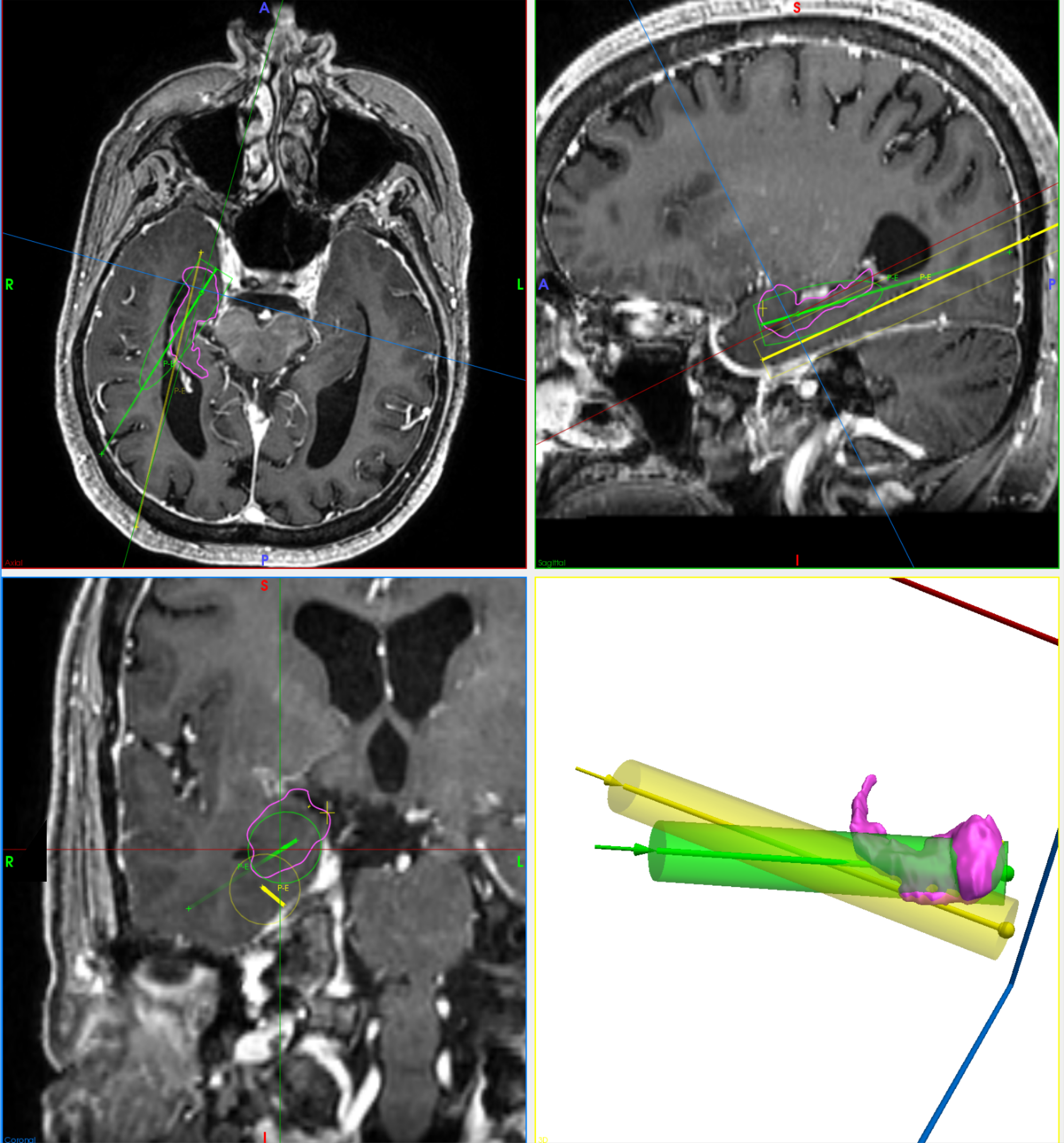

Supplement: Supplementary file 1 [file EPI-60-1949-s001.tif]
