# Supplementary material for: Multicenter validation of automated trajectories for selective laser amygdalohippocampectomy
Source: Epilepsia. 2019 Aug 7;60(9):1949–59. doi: 10.1111/epi.16307 (PMC6771574; doi:10.1111/epi.16307)
Supplement: Supplementary file 2 [file EPI-60-1949-s002.docx]

Supplementary Table 1: Post-hoc analysis of trajectory metrics with Bonferroni correction applied

|  |  | 1 | 2 | 3 | 4 |
| --- | --- | --- | --- | --- | --- |
| Length (mm) | 1 | - | p<0.000 | p<0.000 | p<0.000 |
|  | 2 | p<0.000 | - | p=0.367 | p=0.003 |
|  | 3 | p<0.000 | p=0.367 | - | p<0.000 |
|  | 4 | p<0.000 | p=0.003 | p<0.000 | - |
| Angle (deg) | 1 | - | p=1 | p=1 | p=0.047 |
|  | 2 | p=1 | - | p=1 | p=0.009 |
|  | 3 | p=1 | p=1 | - | p=0.010 |
|  | 4 | p=0.047 | p=0.009 | p=0.010 | - |
| Risk score | 1 | - | p<0.000 | p<0.000 | p<0.000 |
|  | 2 | p<0.000 | - | p=1 | p=0.670 |
|  | 3 | p<0.000 | p=1 | - | p=0.280 |
|  | 4 | p<0.000 | p=0.670 | p=0.280 | - |
| Ablation length (mm) | 1 | - | p=0.410 | p<0.000 | p<0.013 |
|  | 2 | p=0.410 | - | p=0.039 | p=1 |
|  | 3 | p<0.000 | p=0.039 | - | 0.874 |
|  | 4 | p<0.013 | p=1 | p=0.874 | - |
| Ablation Volume (mm^3) | 1 | - | p=0.001 | p=1 | p=0.094 |
|  | 2 | p=0.001 | - | p=0.000 | p=1 |
|  | 3 | p=1 | p<0.000 | - | p=0.012 |
|  | 4 | p=0.094 | p=1 | p=0.012 | - |

Supplementary Table 2: Post-hoc analysis of trajectory ablation volumes

|  |  | 1 | 2 | 3 | 4 |
| --- | --- | --- | --- | --- | --- |
| Ablation Length (mm) | 1 | - | p=0.41 | p<0.000 | p=0.013 |
|  | 2 | p=0.41 | - | p=0.039 | 1 |
|  | 3 | p<0.000 | p=0.039 | - | p=0.74 |
|  | 4 | p=0.013 | p=1 | p=0.74 | - |
| Total ablation volume (mm^3) | 1 | - | p=0.001 | p=1 | p=0.09 |
|  | 2 | p=0.001 | - | p<0.000 | p=1 |
|  | 3 | p=1 | p<0.000 | - | p=0.012 |
|  | 4 | p=0.09 | p=1 | p=0.012 | - |
| Amygdala ablation (%) | 1 | - | p=1 | p<0.000 | p<0.000 |
|  | 2 | p=1 | - | p<0.000 | p<0.000 |
|  | 3 | p<0.000 | p<0.000 | - | p=0.211 |
|  | 4 | p<0.000 | p<0.000 | p=0.211 | - |
| Hippocampal ablation (%) | 1 | - | p=1 | p=1 | p=0.044 |
|  | 2 | p=1 | - | p=1 | 0.557 |
|  | 3 | p=1 | p=1 | - | 0.016 |
|  | 4 | 0.044 | 0.557 | p=0.016 | - |
| Entorhinal cortex ablation (%) | 1 | - | p<0.000 | p<0.000 | p<0.000 |
|  | 2 | p<0.000 | - | p=0.008 | p<0.000 |
|  | 3 | p=0.008 | p=0.008 | - | p=1 |
|  | 4 | p<0.000 | p<0.000 | p=1 | - |
| Parahippocampal ablation (%) | 1 | - | p=0.002 | p=0.953 | p<0.000 |
|  | 2 | p=0.002 | - | p<0.000 | 0.046 |
|  | 3 | p=0.953 | p<0.000 | - | p<0.000 |
|  | 4 | p<0.000 | p=0.046 | p<0.000 | - |

Supplementary Table 3: Mixed Effects Logistic Regression Model of Trajectory Feasibility Ratings (1-4) from Raters (A-C):

| Variable | Odds Ratio Estimate | 95% Confidence Interval |
| --- | --- | --- |
| Trajectory 1 (reference group) | 1 | N/A |
| Trajectory 2 | 2.46 | (1.47, 4.13) |
| Trajectory 3 | 3.08 | (1.83, 5.17) |
| Trajectory 4 | 2.89 | (1.72, 4.85) |
|  |  |  |
| Rater A (reference group) | 1 | N/A |
| Rater B | 3.53 | (2.25, 5.53) |
| Rater C | 5.21 | (3.30, 8.23) |

*Note, odds refers to the odds of a trajectory being rated feasible.

Supplementary Table 4: Estimated frequency of rater preferences and estimated probabilities by trajectory generation method, from the fitted ordinal logistic regression model:

|  | Rater preference  Count (Estimated probability*) | | | |  |
| --- | --- | --- | --- | --- | --- |
| Trajectory | 1 | 2 | 3 | 4 | Total |
| 1 | 6 (0.04) | 4 (0.09) | 6 (0.25) | 32 (0.62) | 48 |
| 2 | 14 (0.30) | 12 (0.29) | 19 (0.26) | 3 (0.14) | 48 |
| 3 | 19 (0.40) | 14 (0.29) | 11 (0.21) | 4 (0.21) | 48 |
| 4 | 9 (0.24) | 18 (0.28) | 12 (0.30) | 9 (0.30) | 48 |
| Total | 48 | 48 | 48 | 48 |  |

*Estimated probabilities of rank for each method as predicted by the ordinal logistic regression model.
